# Direct Reconstruction of High-Fidelity Electrocardiogram Signals From Vector-Based PDF Files With Integrated Deep Learning for Multiparameter Estimation: Retrospective Methodological Study

**DOI:** 10.2196/80597

**Published:** 2026-07-24

**Authors:** Shian-Sen Shie, Po-Yen Huang, Ming-Shien Wen, Chao-Yung Wang

**Affiliations:** 1 Division of Infectious Diseases, Chang Gung Memorial Hospital, Linkou Medical Center, Taoyuan, Taiwan; 2 School of Medicine, College of Medicine, Chang Gung University, Taoyuan, Taiwan; 3 School of Medicine, National Tsing Hua University, Hsinchu, Taiwan; 4 Division of Cardiology, Chang Gung Memorial Hospital, Linkou Medical Center, 5 Fuxing Street, Guishan District, Taoyuan, 33305, Taiwan, 886 33281200 ext 8118; 5 Institute of Cellular and System Medicine, National Health Research Institutes, Zhunan, Taiwan; 6 Department of Medical Science, National Tsing Hua University, Hsinchu, Taiwan

**Keywords:** electrocardiogram, ECG, vector-based PDF, PDF signal reconstruction, deep learning, ECG parameter estimation, multitask learning

## Abstract

**Background:**

Electrocardiograms (ECGs) are commonly stored in PDF, particularly as vector-based files generated by ECG management systems. Previous studies have demonstrated that ECG signals can be extracted through PDF-to-Scalable Vector Graphics (SVG) conversion, highlighting the potential to reconstruct waveform signals from vector graphics. These reconstructed signals further enable the derivation and prediction of clinically relevant ECG parameters.

**Objective:**

This study aimed to develop an integrated framework for direct reconstruction of high-fidelity ECG signals from vector-based PDF files and for simultaneous estimation of multiple clinically relevant ECG parameters using deep learning.

**Methods:**

In this retrospective methodological study, 50,000 twelve-lead ECG PDFs generated by a MUSE system (2015‐2024) were analyzed. A direct PDF parsing pipeline was developed to extract vector path objects and reconstruct time-series signals without intermediate format conversion. Reconstruction accuracy was evaluated against the original system-exported signals. Two deep learning models, DualECGFormer and DualResNetECG, were developed to estimate 8 specific ECG parameters. The reference standards for these parameters—including ventricular rate; PR interval; QRS duration; QT interval; corrected QT interval (QTc); and the electrical axes of the P wave, QRS complex, and T wave—were derived from machine-generated values within the MUSE system database. Performance was compared with a rule-based approach (NeuroKit2).

**Results:**

The proposed method achieved high reconstruction fidelity, with mean absolute errors (MAEs) below 1×10^–3^ mV across all leads. Compared with an SVG-based workflow, the direct parsing approach reduced processing time by approximately 4.6-fold. For parameter estimation, deep learning models outperformed the rule-based method for most parameters. DualResNetECG achieved the best overall performance, with MAEs of 1.11 bpm for ventricular rate, 7.27 milliseconds for PR interval, 9.89 milliseconds for QRS duration, 11.87 milliseconds for QT, and 13.71 milliseconds for QTc. For electrical axis estimation, MAEs ranged from 8.0° to 16.31°. The model also demonstrated reliable detection of physiologically undefined parameters (PR interval and P-wave axis), achieving an area under the receiver operating characteristic curve of up to 0.978.

**Conclusions:**

This study presents an efficient and scalable framework for direct extraction of ECG signals from MUSE-generated vector-based PDFs and integrated multiparameter estimation using deep learning. The approach achieves high reconstruction accuracy and competitive predictive performance, supporting its potential utility for large-scale retrospective MUSE ECG analysis.

## Introduction

The extraction of time-series data from electrocardiograms (ECGs) is of critical importance for clinical diagnosis and cardiology research. In contemporary clinical workflows, PDF has become the predominant medium for ECG storage and exchange due to its cross-platform compatibility [1]. ECG PDFs can generally be categorized into 2 types: rasterized images generated through scanning or photography and vector-based documents directly produced by ECG management systems (eg, MUSE; GE HealthCare).

For rasterized ECGs lacking internal structural information, existing studies have primarily focused on image-processing–based approaches, including grid-line suppression, morphological filtering, and end-to-end deep learning models for waveform reconstruction [2-6]. While these methods have demonstrated utility in digitizing paper-based ECGs, they are computationally intensive and highly susceptible to image noise and resolution variability, which may compromise signal segmentation and reconstruction accuracy [7].

In contrast, vector-based ECG PDFs containing embedded graphical objects provide an opportunity for direct digital signal extraction. One commonly adopted approach involves converting PDFs into Scalable Vector Graphics (SVG) format, followed by parsing path elements to reconstruct time-series signals [8]. However, this transformation pipeline introduces additional intermediate software layers, thereby increasing workflow complexity and limiting efficiency in large-scale clinical data processing. Beyond signal extraction, the estimation of clinically relevant parameters—including ventricular rate; PR interval; QRS duration; QT interval and corrected QT interval (QTc); and the electrical axes of the P wave, QRS complex, and T wave—constitutes a central component of ECG interpretation. Current approaches primarily rely on rule-based signal processing toolkits (eg, NeuroKit2) [9] or deep learning–based regression models, with the latter generally demonstrating superior performance [10]. However, most deep learning models focus on predicting only a limited subset of parameters (eg, QT and QTc intervals) and lack integrative design, making it difficult to provide comprehensive ECG diagnostic information [11-14]. In particular, simultaneous estimation of electrical axis parameters (P-, R-, and T-wave axes) remains relatively underexplored.

To address these limitations, we propose a streamlined and integrative framework specifically optimized for MUSE-generated vector-based ECG PDFs. First, we developed a method to directly parse embedded path objects from these vector-based PDFs, thereby eliminating the need for intermediate format conversion and enabling rapid reconstruction of ECG time-series signals. Second, leveraging these reconstructed signals, we developed a multitask deep learning model capable of the simultaneous estimation of 8 critical clinical parameters: ventricular rate; PR interval; QRS duration; QT; QTc; and the electrical axes (P, R, and T). By consolidating high-fidelity signal extraction with comprehensive parameter prediction into a single pipeline, this study provided a framework for ECG digitization and parameter estimation, offering a more integrated solution than currently available segmented approaches.

## Methods

### Study Design and Data Source

This retrospective methodological study was designed to establish a pipeline for directly reconstructing time-series signals from ECG PDF files and to further develop a deep learning model for ECG parameter estimation. The study dataset consisted of 12-lead ECG PDFs generated from the MUSE ECG management system (GE HealthCare) at our institution. All ECGs were recorded using the standard 3×4+1 layout, comprising limb leads (I, II, III), augmented limb leads (aVR, aVL, aVF), precordial leads (V1-V6), and an extended long lead II for rhythm monitoring. The study period spanned from 2015 to 2024, during which a total of 50,000 ECG PDFs were collected for analysis. For model development, 45,000 (90%) ECGs were allocated and randomly split into a training dataset (80%) and a validation dataset (20%). An additional 5000 ECGs were randomly selected and reserved as an independent testing dataset for final performance evaluation. Dataset partitioning was performed at the patient level to prevent ECG recordings from the same individual from appearing across multiple datasets, thereby minimizing potential data leakage.

### Ethical Considerations

The study protocol for “analysis of electrocardiograms using deep learning” was reviewed and approved by the institutional review board (IRB) of Linkou Chang Gung Memorial Hospital (202301533B0) and was conducted in accordance with the Declaration of Helsinki. All ECG data were fully anonymized prior to analysis to ensure patient confidentiality and compliance with ethical standards. The requirement for informed consent was waived by the IRB because of the retrospective nature of the study and the use of fully anonymized data.

### ECG PDF Parsing and Signal Reconstruction

#### PDF Analysis Pipeline

**Figure 1.**
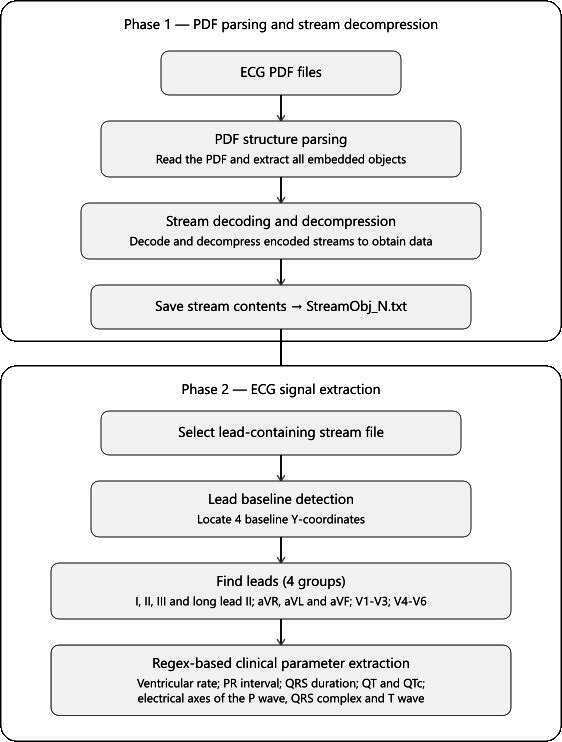
Workflow illustrating the reconstruction of electrocardiogram (ECG) time-series signals from PDF files and the extraction of 8 machine-generated clinical parameters from the embedded textual annotations, including ventricular rate; PR interval; QRS duration; QT; corrected QT interval (QTc); and electrical axes of the P wave, QRS complex, and T wave.

The data processing workflow for this study is illustrated in Figure 1. We integrated components of PdfFileAnalyzer, an open-source C#-based project, into our processing pipeline to analyze the ECG PDF files [15]. PdfFileAnalyzer is specifically designed to read, parse, and display the internal structure of PDF documents. Through this process, each PDF was decomposed into multiple text-based representations of its constituent objects. Among these, the stream object files contained the critical coordinate information of the ECG waveforms embedded within the vector graphics. By decoding this specific file, coordinate data were extracted and subsequently transformed into time-series representations of time and voltage using predefined scaling formulas.

**Figure 2.**
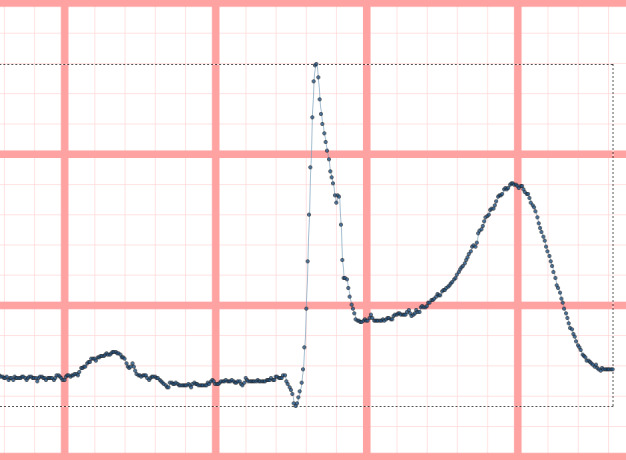
Electrocardiogram waveforms are represented as path objects, which are composed of numerous points connected to form continuous line segments.

Detailed examination of the internal PDF structure revealed that ECG waveforms are encoded as vector-based path objects, consisting of sequential coordinate points connected by drawing commands to form continuous line segments (Figure 2). Exploiting this characteristic enabled accurate reconstruction of signals that closely approximate the original ECG waveforms. These vector representations are stored within *StreamObj_N.txt* files, which contain sequences of Cartesian coordinates associated with PDF drawing commands, such as *Move-to* (M), *Line-to* (L), and *Stroke* (S), defining the geometric paths of each ECG lead. A representative excerpt for lead I, together with the interpretation of the associated commands, is presented in Multimedia Appendix 1.

In parallel with waveform reconstruction, the PDF parsing process also enabled extraction of 8 machine-generated clinical parameters embedded as textual annotations within the ECG report, including ventricular rate; PR interval; QRS duration; QT interval; QTc; and the electrical axes of the P wave, QRS complex, and T wave. These parameters, automatically generated by the ECG acquisition system, were used as the reference standards (ground truth labels) for both the rule-based analyses and the training and evaluation of the deep learning models. The labels were derived from the automated interpretations produced by the MUSE ECG management system rather than from manual adjudications by expert cardiologists. Although not clinically validated by cardiologists, these labels provided a consistent benchmark for evaluating the models’ ability to reproduce the diagnostic outputs of the original ECG acquisition system.

Notably, under certain cardiac rhythm abnormalities—such as atrial fibrillation and junctional rhythm—specific parameters, particularly the PR interval and P-wave axis, are physiologically undefined due to the absence or inconsistency of discernible P waves. In such cases, these parameters are reported as missing values in the system-generated ECG report. This characteristic was explicitly incorporated into the proposed deep learning framework and leveraged during both training and inference, enabling the model to appropriately account for physiologically undefined parameters.

#### Coordinate Transformation for ECG Signal Reconstruction

**Figure 3.**
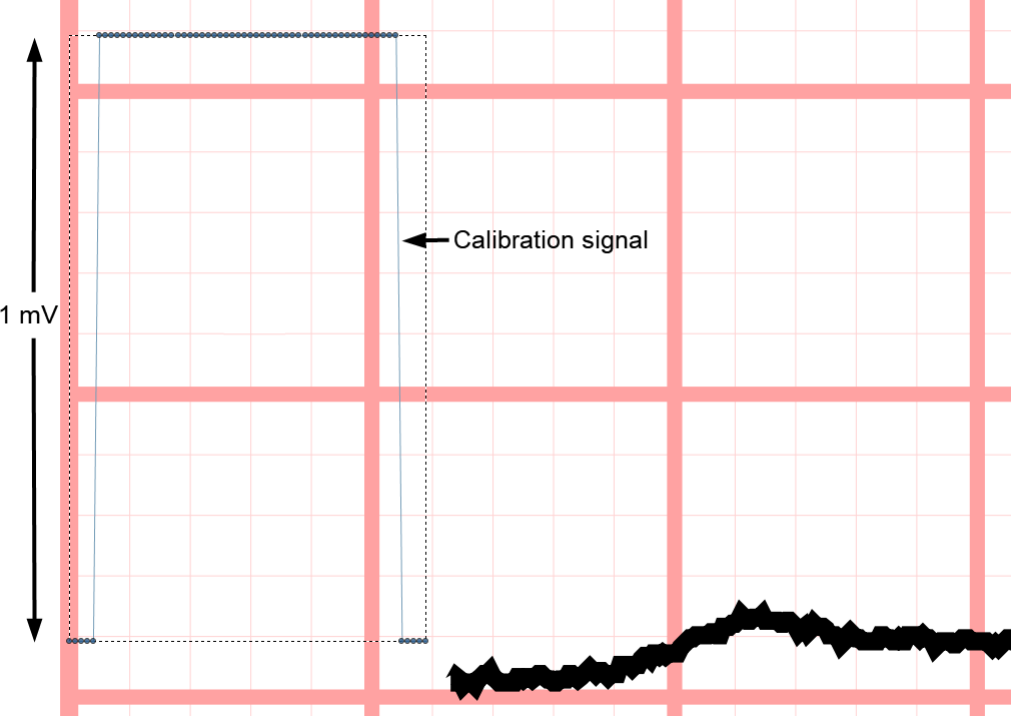
Calibration signal for lead I located on the left side of the electrocardiogram tracing. The calibration signal is represented by a path object comprising 60 points. The height of this object corresponds to 1 mV, with y-axis coordinates ranging from a minimum of 14,242 to a maximum of 15,242.

In the PDF coordinate system, the origin (0,0) is located at the bottom-left corner. Each ECG lead includes a 1 mV calibration signal on the left side, which can be identified as a distinct path object (Figure 3). In this study, the calibration signal corresponds to a vertical span of 1000 y-axis units, yielding a voltage scaling factor of 0.001 mV per unit.

The long lead II path object spans 10 seconds and contains 5000 waveform points, corresponding to a sampling rate of 500 Hz. In the PDF, this path object is represented by exactly 25,000 x-axis units, yielding a horizontal scaling factor of 0.0004 seconds per unit, which is used to convert the x-coordinates into time values for the reconstructed waveform.

On the basis of the above analysis, the time and voltage of each point within the path object can be determined using the following formulas:


(1) $$Time=\left(x-X_{start}\right)\times 0.0004\sec$$

(2) $$Voltage=\left(y-Y_{start}\right)\times 0.001\;mV$$

The variables $X_{start}$ and $Y_{start}$ represent the x-coordinate and y-coordinate of the starting point of each lead’s waveform within the PDF, respectively. The starting points for all leads are summarized in Table 1.

**Table 1.** Starting x and y coordinates for each lead’s signal^a^.

Lead	X_start_(coordinate)	Y_start_(coordinate)
I	1080	14,242
II	1080	10,545
III	1080	6848
aVR	7330	14,242
aVF	7330	10,545
aVL	7330	6848
V1	13,580	14,242
V2	13,580	10,545
V3	13,580	6848
V4	19,830	14,242
V5	19,830	10,545
V6	19,830	6848
Long II	1080	3150

a Note that these values are provided as examples and may vary depending on the specific PDF file. The x and y values represent the coordinates of path points in the PDF user coordinate system extracted from the vector-based PDF.

The overall procedure for processing and extracting ECG information from PDF files is summarized in pseudocode form (Textbox 1). The pseudocode outlines the key steps, including PDF parsing, identification of waveform and reference voltage path objects, coordinate extraction, scaling factor estimation, and transformation into time-series signals for each ECG lead. For comparative evaluation, ECG PDF files were converted to SVG format using Inkscape (version 1.4.3), and the resulting vectorized graphical elements were parsed to reconstruct ECG time-series signals for direct comparison with the proposed method.

Textbox 1. **Pseudocode summarizing the workflow for electrocardiogram (ECG) waveform reconstruction and extraction of machine-generated clinical parameters from MUSE ECG PDF files.**

INPUT:

SelectedFolder – Directory containing ECG PDF files

OUTPUT:

Reconstructed 12-lead ECG time-series signals and associated clinical parameters

- initialize SelectedFolder containing ECG PDF files;
- for each PDF document do
  - Read and parse the PDF file;
  - Decompress PDF into multiple stream objects;
  - Select the largest stream object containing waveform data;
  - ecg ← ECG(streamFile);
  - Save reconstructed ECG signals and clinical parameters;

ECG(streamFile):

- bases ← FindLeadBase(streamFile);
- FindLead(startX=1080, bases, leads=[I, II, III, longII]);
- FindLead(startX=7330, bases, leads=[aVR, aVL, aVF]);
- FindLead(startX=13580, bases, leads=[V1, V2, V3]);
- FindLead(startX=19830, bases, leads=[V4, V5, V6]);
- Extract clinical parameters from streamFile:
  - Ventricular rate;
  - PR interval;
  - QRS duration;
  - QT/QTc interval;
  - P, R, T axes;
- return ECG object;

FindLeadBase(streamFile):

- for each line matching “450 (Y) m” do
  - collect unique Y values;
  - stop when four baseline positions are identified;
- return bases;

FindLead(startX, bases[], leads[]):

- for each lead i do
  - locate line matching “(startX Y m)” as waveform origin;
  - initialize time = 0;
  - compute voltage = (Y - bases[i]);
  - while next line matches “(X Y l)” do
    - compute time = (X - startX);
    - compute voltage = (Y - bases[i]);
    - append (time, voltage) to leads[i];

To evaluate the accuracy of the proposed signal reconstruction process, the ECG time-series data restored from the PDF files were cross-compared with the original ECG signals exported directly from the underlying MUSE system. The assessment focused on the consistency of sampling frequency and differences in voltage amplitude between the 2 datasets. Quantitative evaluation was performed using standard statistical metrics, including mean absolute error (MAE) with corresponding CIs, as well as correlation and agreement analyses.

### Estimation of Clinical ECG Parameters Using Rule-Based and Deep Learning Approaches

ECG data reconstructed from PDF files were analyzed, including standard 12-lead recordings (2.5 s each) and a 10-second long lead II. Three different approaches were applied to estimate 8 clinical ECG parameters: ventricular rate; PR interval; QRS duration; QT interval; QTc; and the electrical axes of the P wave, QRS complex, and T wave.

#### Rule-Based Parameter Estimation

The rule-based parameter estimation pipeline was developed using the open-source biomedical signal processing library NeuroKit2 [9]. The long lead II was selected as the primary signal source due to its suitability for rhythm analysis. Preprocessing steps included noise reduction and baseline wander removal implemented through NeuroKit2’s standardized filtering procedures. R-peak detection was performed using a multialgorithm strategy incorporating the NeuroKit, Pan-Tompkins, and Nabian methods to enhance detection robustness [9,16,17]. Ventricular rate was calculated from the mean R-R interval when at least 2 R peaks were successfully identified. Wave delineation was achieved using a discrete wavelet transform–based approach to identify key fiducial points, including the onset of the P wave, the onset and offset of the QRS complex, and the end of the T wave. These delineations enabled the computation of the PR interval, QRS duration, and QT interval, all expressed in milliseconds. The QTc was subsequently calculated using Bazett formula. Electrical axis estimation was based on frontal plane vector analysis utilizing signals from leads I and aVF. Representative amplitudes of the P wave, QRS complex, and T wave were determined for each lead, and the corresponding electrical axes were calculated using the arctangent function, yielding angular measurements in degrees.

Overall, this rule-based pipeline provides an interpretable and standardized approach for ECG parameter estimation and serves as a benchmark for comparison with the proposed deep learning models.

#### Deep Learning Models for ECG Parameter Estimation

Both proposed deep learning models—DualECGFormer and DualResNetECG—adopt a dual-input configuration to process reconstructed ECG signals. Inputs consist of standard 12-lead ECG signals (2.5 s each) and a long lead II signal (10 s). Both models generate 8 independent outputs corresponding to ventricular rate; PR interval; QRS duration; QT interval; QTc; and the electrical axes of the P wave, QRS complex, and T wave. For each parameter, two types of outputs are produced:

1. Value prediction—a regression output representing the numerical estimate of the parameter.
2. Existence prediction—a binary classification output indicating whether the parameter is defined in the reference ECG report. This design accommodates cases in which certain parameters, such as the PR interval or P-wave axis, may be physiologically undefined.

#### Data Preprocessing and Input Preparation

ECG waveforms were directly reconstructed from ECG PDF files without additional signal enhancement procedures, such as filtering, denoising, baseline wander correction, or global amplitude normalization. The reconstructed signals were used as direct model inputs to preserve the original waveform morphology encoded within the source PDF files. To ensure consistent input dimensionality across all recordings, standard 12-lead ECG signals (2.5 s per lead) were truncated or zero-padded to 1250 sampling points, whereas long lead II recordings (10 s) were adjusted to 5000 sampling points. Continuous ECG parameter labels were normalized using *z* score normalization based on the mean and SD estimated exclusively from the training dataset. Normalization statistics were independently computed for each ECG parameter using valid nonmissing samples. Inverse normalization was subsequently applied during inference and evaluation to restore predictions to their original clinical units.

#### DualECGFormer: Convolutional Neural Network-Transformer Backbone

**Figure 4.**
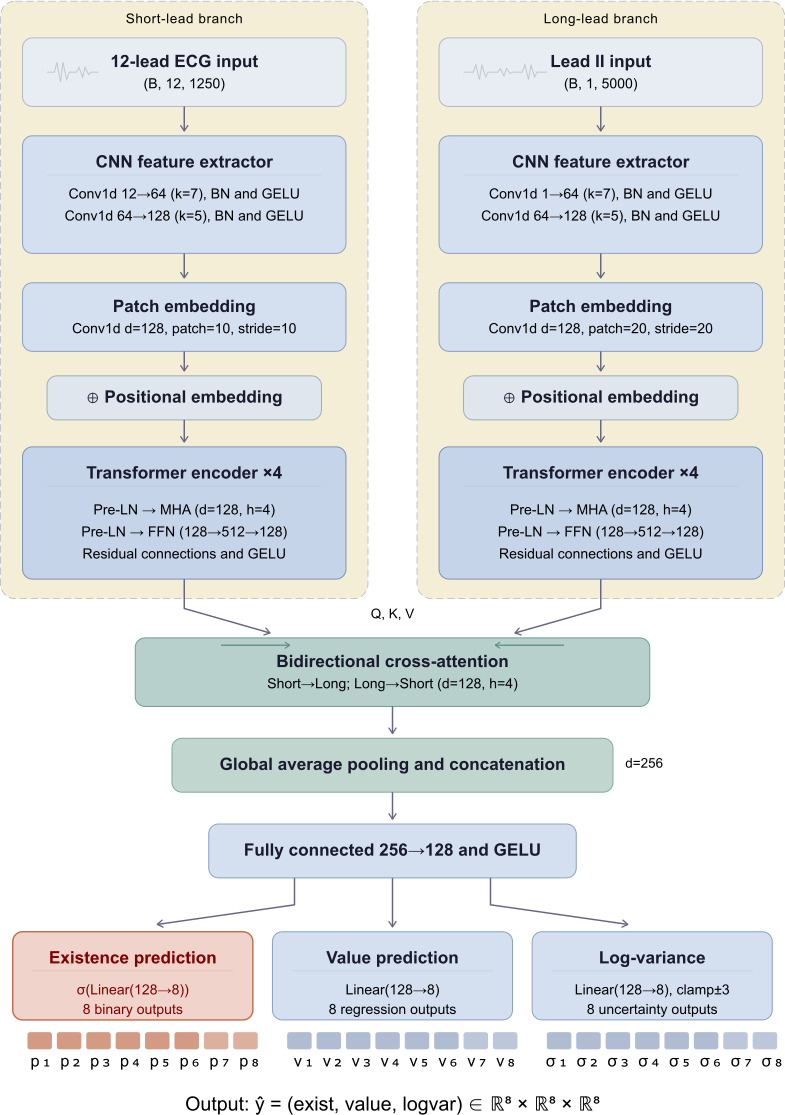
Architecture of the DualECGFormer model. BN: batch normalization; CNN: convolutional neural network; Conv1d: one-dimensional convolution; ECG: electrocardiogram; FFN: feed-forward network; GELU: Gaussian error linear unit; MHA: multihead attention; Pre-LN: prelayer normalization.

The DualECGFormer model uses a hybrid convolutional neural network (CNN)-Transformer architecture (Figure 4) [18]. Each input branch is initially processed through a ConvFeature module composed of 1D convolutional layers (kernel size 7, padding 3), followed by batch normalization and Gaussian error linear unit activation. Extracted features are projected into sequential embeddings using a PatchEmbed1D module (kernel size=stride=10), producing token sequences of dimension d_model_=128.

The embedded sequences are processed by 4 stacked Transformer blocks (depth=4). Each block contains a multihead self-attention mechanism (d_head_=4) to model temporal dependencies, a position-wise feed-forward network, layer normalization, residual connections, and positional embeddings. These components collectively encode both local and long-range interactions within the ECG signal.

#### DualResNetECG: Squeeze-and-Excitation Residual Network Backbone

**Figure 5.**
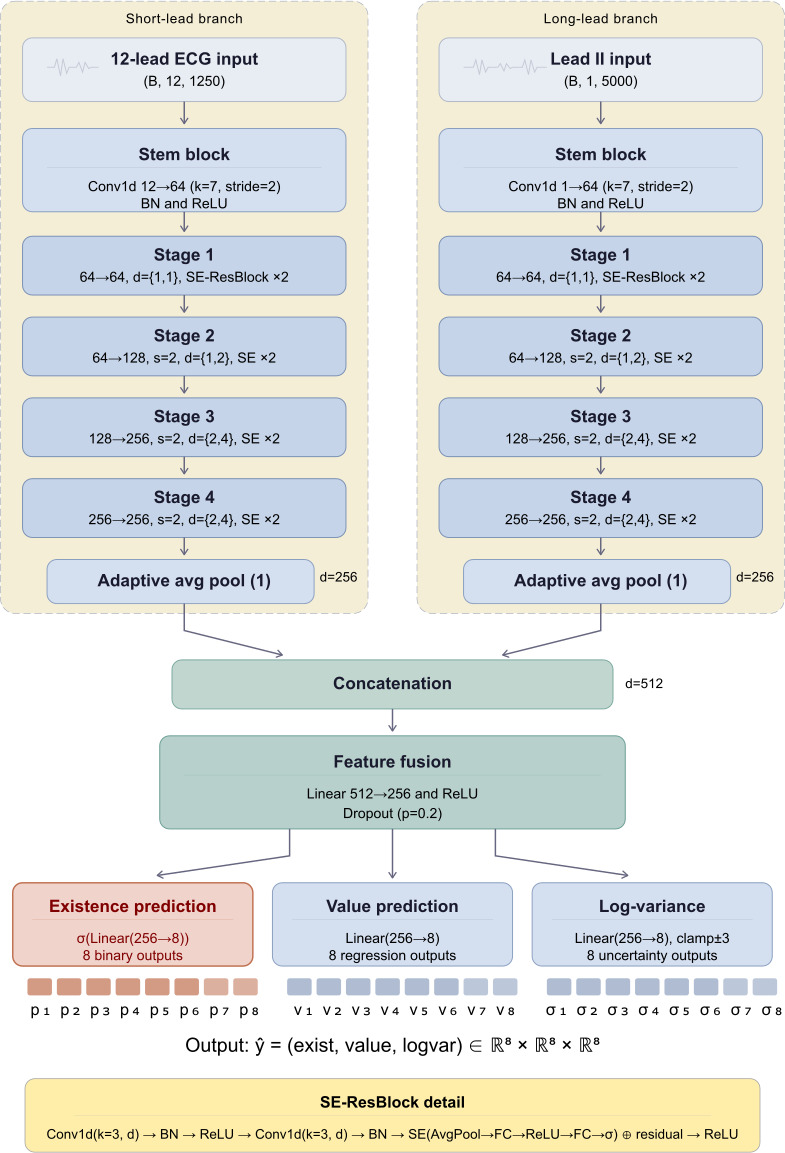
Architecture of the DualResNetECG model. AvgPool: average pooling; BN: batch normalization; Conv1d: one-dimensional convolution; ECG: electrocardiogram; FC: fully connected; ReLU: rectified linear unit; ResBlock: residual block; SE: Squeeze-and-Excitation; σ: sigmoid activation function.

The DualResNetECG model uses a 1D residual network (ResNet1D) backbone augmented with Squeeze-and-Excitation (SE) modules (Figure 5) [19,20]. Each input branch passes through an independent ResNet1D, with the basic building unit being a 1D residual block (ResBlock1D). SE modules perform global average pooling along the temporal dimension and use 2 fully connected layers (reduction ratio=8) to achieve channel-wise feature recalibration, modulating the relative importance of different feature channels.

Feature representations from the dual branches are fused and passed to task-specific prediction heads. The multitask output design is identical to that of DualECGFormer, providing both value and existence predictions for each of the 8 ECG parameters.

#### Loss Function and Optimization Strategy

Both DualECGFormer and DualResNetECG are trained using a composite loss function that jointly optimizes regression and classification objectives. The loss function is formulated as a weighted combination of:

- Binary cross-entropy loss: applied to the existence prediction outputs to determine whether each ECG parameter is defined.
- Heteroscedastic regression loss: applied to the value prediction outputs, incorporating uncertainty weighting to account for varying confidence across parameters and missing labels.

Formally, the total loss *L* is expressed as:


$$L=\lambda_{\text{exist}}\cdot L_{\text{BCE}}+\lambda_{\text{value}}\cdot L_{\text{regression}}$$

where *λ*_*exist*_ and *λ*_*value*_ are dynamic weighting factors. This approach ensures balanced optimization between estimation of ECG parameter values and prediction of their presence or absence in physiologically undefined cases.

#### Model Training and Implementation Details

Model training was performed using the Adam optimizer with an initial learning rate of 1×10^−4^, a batch size of 256, and a maximum of 100 epochs. Early stopping with a patience of 10 epochs was applied based on validation performance to reduce overfitting. Hyperparameters were empirically selected according to preliminary validation experiments. All experiments were implemented using PyTorch (version 2.7.0; Meta AI) with CUDA (Compute Unified Device Architecture; version 12.8; NVIDIA Corporation) and conducted on a workstation equipped with an NVIDIA RTX 5080 graphics processing unit (GPU). Random seeds were fixed at 42 to improve experimental reproducibility.

## Results

### Accuracy of ECG Signal Reconstruction

**Figure 6.**
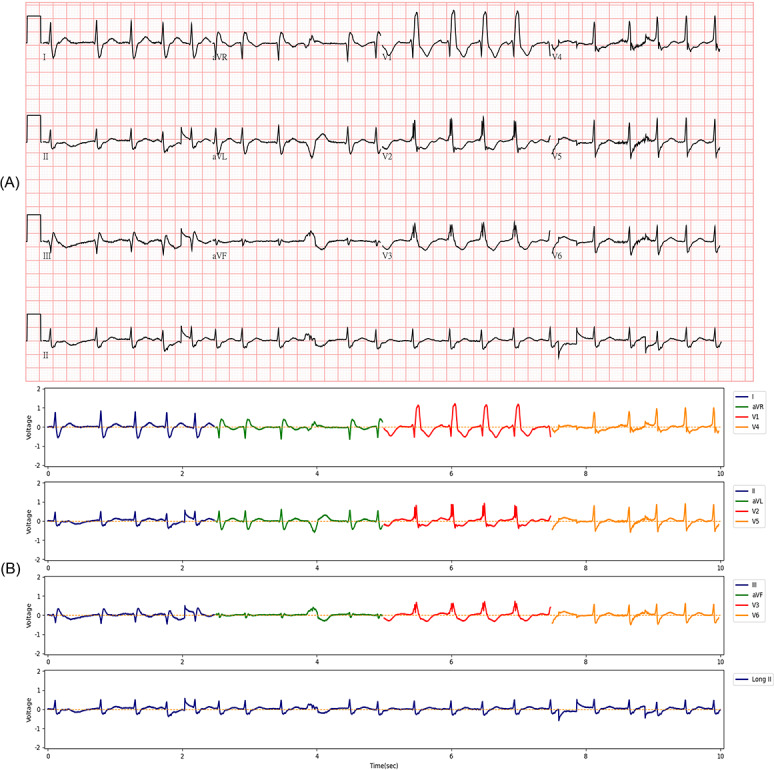
(A) Original electrocardiogram (ECG) image from the PDF file. (B) Reconstructed 12-lead ECG waveforms generated from the extracted time-series data. The reconstructed signals closely match the original ECG, demonstrating high visual and morphological agreement.

The accuracy of the proposed ECG signal reconstruction method was evaluated by comparing the time-series signals derived from PDF files with the original signals exported directly from the MUSE system. As summarized in Table 2, the reconstruction errors were minimal across all leads. The MAE ranged from 2.55×10^–4^ to 2.69×10^–4^ mV, with narrow 95% CIs, indicating high consistency between reconstructed and reference signals. Pearson correlation coefficients were uniformly 0.99 for all leads, demonstrating a consistent linear relationship. Bland-Altman analysis revealed negligible systematic bias, with mean differences close to 0 (on the order of 10^–6^ mV). The limits of agreement were consistently within approximately +7.0×10^–4^ mV to –7.0×10^–4^ mV, suggesting close agreement between the 2 datasets. Comparable performance was observed across limb leads, augmented leads, precordial leads, and the long lead II. A representative example of the reconstructed 12-lead ECG is shown in Figure 6, demonstrating visual concordance with the original waveform and preservation of its morphological and structural characteristics.

**Table 2.** Perlead reconstruction accuracy of electrocardiogram signals derived from PDF files compared with original MUSE system data^a^.

Lead	Mean absolute error (95% CI)	Pearson *r*	Bias	Limits of agreement
I	2.61×10^–4^ (2.47×10^–4^ to 2.76×10^–4^)	0.99	1.92×10^–6^	–7.03×10^–4^ to 7.07×10^–4^
II	2.55×10^–4^ (2.43×10^–4^ to 2.67×10^–4^)	0.99	–1.35×10^–6^	–6.85×10^–4^ to 6.82×10^–4^
III	2.64×10^–4^ (2.51×10^–4^ to 2.74×10^–4^)	0.99	1.96×10^–6^	–7.08×10^–4^ to 7.12×10^–4^
aVR	2.65×10^–4^ (2.49×10^–4^ to 2.79×10^–4^)	0.99	1.29×10^–6^	–7.11×10^–4^ to 7.14×10^–4^
aVL	2.68×10^–4^ (2.55×10^–4^ to 2.8×10^–4^)	0.99	–3.97×10^–6^	–7.23×10^–4^ to 7.15×10^–4^
aVF	2.58×10^–4^ (2.46×10^–4^ to 2.71×10^–4^)	0.99	3.23×10^–6^	–6.93×10^–4^ to 7.01×10^–4^
V1	2.67×10^–4^ (2.54×10^–4^ to 2.81×10^–4^)	0.99	–6.67×10^–7^	–7.21×10^–4^ to 7.19×10^–4^
V2	2.66×10^–4^ (2.51×10^–4^ to 2.81×10^–4^)	0.99	–1.49×10^–6^	–7.19×10^–4^ to 7.16×10^–4^
V3	2.68×10⁻⁴ (2.55×10^–4^ to 2.8×10^–4^)	0.99	–1.32×10^–6^	–7.22×10^–4^ to 7.19×10^–4^
V4	2.59×10^–4^ (2.43×10^–4^ to 2.74×10^–4^)	0.99	–4.67×10^–7^	–6.98×10^–4^ to 6.97×10^–4^
V5	2.61×10^–4^ (2.46×10^–4^ to 2.77×10^–4^)	0.99	–1.21×10^–6^	–7.07×10^–4^ to 7.05×10^–4^
V6	2.69×10^–4^ (2.55×10^–4^ to 2.82×10^–4^)	0.99	–2.58×10^–6^	–7.29×10^–4^ to 7.24×10^–4^
Long II	2.68×10^–4^ (2.56×10^–4^ to 2.81×10^–4^)	0.99	–3.97×10^–7^	–7.24×10^–4^ to 7.22×10^–4^

a All values are expressed in mV.

Both the proposed direct PDF parsing method and the PDF-to-SVG–based workflow achieved a 100% success rate in ECG waveform reconstruction. The reconstructed signals from both approaches were identical in terms of the number of extracted samples and their corresponding voltage values. However, significant differences in computational efficiency were observed. When processing a total of 50,000 ECG PDF files, the direct PDF parsing method required 445 minutes, whereas the SVG-based workflow required 2029 minutes, representing an approximately 4.6-fold increase in processing time.

### Performance of ECG Parameter Estimation

#### Overview

The performance of the rule-based approach (NeuroKit2) and the 2 deep learning models (DualECGFormer and DualResNetECG) for estimating 8 ECG clinical parameters is presented in Table 3.

**Table 3.** Performance comparison for electrocardiogram parameter estimation across 3 methods.

Parameter and metric	NeuroKit2	DualECGFormer	DualResNetECG
Ventricular rate (bpm)			
MAE^a^ (95% CI)	1.18 (1.01 to 1.35)	3.87 (3.67 to 4.07)	1.11 (1.03 to 1.21)
RMSE^b^ (95% CI)	5.92 (4.91 to 6.92)	7.82 (7.06 to 8.72)	3.48 (2.52 to 4.37)
*R*^2c^(95% CI)	0.88 (0.84 to 0.92)	0.87 (0.84 to 0.90)	0.97 (0.96 to 0.99)
PR interval (ms)			
MAE (95% CI)	58.99 (57.99 to 59.97)	18.30 (17.76 to 18.87)	7.27 (6.92 to 7.65)
RMSE (95% CI)	67.65 (66.64 to 68.74)	26.48 (24.95 to 27.94)	14.28 (12.55 to 16.29)
*R*^2^ (95% CI)	−4.73 (−5.3 to –4.26)	0.21 (0.18 to 0.23)	0.77 (0.72 to 0.81)
QRS duration (ms)			
MAE (95% CI)	38.03 (36.92 to 39.21)	20.24 (19.62 to 20.87)	9.89 (9.41 to 10.42)
RMSE (95% CI)	53.1 (51.33 to 55.04)	29.93 (28.51 to 31.51)	20.83 (19.06 to 22.51)
*R*^2^ (95% CI)	−2.53 (−2.92 to –2.21)	0.29 (0.25 to 0.33)	0.65 (0.61 to 0.70)
QT interval (ms)			
MAE (95% CI)	54.63 (53.28 to 56.03)	21.11 (20.38 to 21.80)	11.87 (11.31 to 12.45)
RMSE (95% CI)	71.79 (69.51 to 74.13)	34.36 (31.98 to 36.84)	23.10 (21.05 to 25.35)
*R*^2^ (95% CI)	−2.16 (-2.45 to –1.9)	0.56 (0.53 to 0.59)	0.80 (0.77 to 0.83)
Corrected QT interval (ms)			
MAE (95% CI)	62.13 (60.62 to 63.64)	23.19 (22.31 to 24.12)	13.71 (13.10 to 14.27)
RMSE (95% CI)	80.03 (77.83 to 82.14)	39.52 (35.98 to 42.92)	25.94 (23.70 to 28.36)
*R*^2^ (95% CI)	−5.23 (−5.68 to –4.85)	0.29 (0.26 to 0.32)	0.70 (0.63 to 0.75)
P-wave axis (°)			
MAE (95% CI)	22.24 (21.74 to 22.76)	14.36 (13.89 to 14.85)	9.33 (8.85 to 9.82)
RMSE (95% CI)	28.28 (27.52 to 29.1)	22.71 (21.16 to 24.36)	18.28 (16.18 to 20.25)
*R*^2^ (95% CI)	−0.53 (−0.61 to –0.45)	0.21 (0.18 to 0.24)	0.49 (0.44 to 0.55)
R-wave axis (°)			
MAE (95% CI)	16.61 (15.53 to 17.77)	11.37 (10.63 to 12.22)	8.0 (7.45 to 8.59)
RMSE (95% CI)	41.81 (37.56 to 46.17)	28.67 (26.17 to 31.30)	22.14 (19.45 to 24.79)
*R*^2^ (95% CI)	0.01 (−0.14 to 0.18)	0.61 (0.56 to 0.66)	0.77 (0.72 to 0.81)
T-wave axis (°)			
MAE (95% CI)	28.62 (27.64 to 29.64)	24.41 (23.43 to 25.43)	16.31 (15.40 to 17.19)
RMSE (95% CI)	44.6 (42.54 to 46.66)	43.04 (41.25 to 44.88)	34.94 (32.91 to 37.02)
*R*^2^ (95% CI)	−0.15 (−0.18 to –0.12)	0.23 (0.20 to 0.26)	0.49 (0.46 to 0.52)

a MAE: mean absolute error.

b RMSE: root mean square error.

c *R*2: coefficient of determination.

#### Ventricular Rate

All 3 methods demonstrated good agreement with the reference standard. DualResNetECG achieved the lowest MAE (1.11 bpm, 95% CI 1.03‐1.21) and RMSE (3.48 bpm, 95% CI 2.52‐4.37), along with the highest coefficient of determination (*R*^2^=0.97, 95% CI 0.96‐0.99). NeuroKit2 showed comparable MAE (1.18 bpm, 95% CI 1.01-1.35) but a higher RMSE (5.92 bpm, 95% CI 4.91-6.92) and lower *R*² (0.88, 95% CI 0.84-0.92). DualECGFormer yielded slightly larger errors (MAE 3.87 bpm, 95% CI 3.67‐4.07) with an *R*^2^ of 0.87 (95% CI 0.84‐0.90).

#### Temporal Intervals (PR, QRS, QT, and QTc)

For the estimation of ECG temporal intervals, both deep learning models outperformed the rule-based approach. DualResNetECG consistently achieved the lowest errors, with MAEs of 7.27 milliseconds (95% CI 6.92‐7.65) for the PR interval, 9.89 milliseconds (95% CI 9.41‐10.42) for the QRS duration, 11.87 milliseconds (95% CI 11.31‐12.45) for the QT interval, and 13.71 milliseconds (95% CI 13.10‐14.27) for the QTc. Corresponding *R*^2^ values ranged from 0.65 (95% CI 0.61-0.70) to 0.80 (95% CI 0.77-0.83), indicating substantial agreement with the reference measurements.

DualECGFormer also demonstrated improved performance compared with NeuroKit2, with MAEs of 18.30 milliseconds (95% CI 17.76-18.87) for the PR interval, 20.24 milliseconds (95% CI 19.62-20.87) for the QRS duration, 21.11 milliseconds (95% CI 20.38-21.80) for the QT interval, and 23.19 milliseconds (95% CI 22.31-24.12) for the QTc, and *R*^2^ values between 0.21 (95% CI 0.18-0.23) and 0.56 (95% CI 0.53-0.59). In contrast, the rule-based NeuroKit2 approach exhibited considerably larger errors, with MAEs ranging from 38.03 milliseconds (95% CI 36.92-39.21) to 62.13 milliseconds (95% CI 60.62-63.64) and negative *R*^2^ values for these parameters, indicating limited agreement with the reference standard.

#### Electrical Axis Estimation

For the estimation of the electrical axes of the P wave, QRS complex (R axis), and T wave, DualResNetECG again achieved the best performance, with MAEs of 9.33° (95% CI 8.85-9.82), 8.0° (95% CI 7.45-8.59), and 16.31° (95% CI 15.40-17.19), respectively, and *R*^2^ values of 0.49 (95% CI 0.44-0.55), 0.77 (95% CI 0.72-0.81), and 0.49 (95% CI 0.46-0.52). DualECGFormer demonstrated intermediate performance, while NeuroKit2 showed higher errors and lower or negative *R*^2^ values, particularly for the P and T axes.

#### Detection of Parameter Existence

As NeuroKit2 was not able to reliably determine the existence of certain physiologically defined parameters—particularly the PR interval and P-wave axis—the performance of the deep learning models in detecting the presence of these parameters is summarized in Table 4. For PR interval existence, DualResNetECG achieved superior performance, with an accuracy of 0.96, precision of 0.98, recall of 0.98, specificity of 0.81, and an *F*_1_-score of 0.98. DualECGFormer also demonstrated strong performance, with an accuracy of 0.93 and an *F*_1_-score of 0.96, although with lower specificity (0.60).

**Table 4.** Performance of DualECGFormer and DualResNetECG for detecting the presence of PR interval and P-wave axis.

Parameter and metric	DualECGFormer	DualResNetECG
PR interval (existence)		
Accuracy	0.93	0.96
Precision	0.95	0.98
Recall	0.97	0.98
Specificity	0.60	0.81
*F*_1_-score	0.96	0.98
NPV^a^	0.69	0.79
P-wave axis (existence)		
Accuracy	0.92	0.95
Precision	0.95	0.98
Recall	0.96	0.97
Specificity	0.63	0.82
*F*_1_-score	0.96	0.97
NPV	0.68	0.79

a NPV: negative predictive value.

Similarly, for P-wave axis existence, DualResNetECG outperformed DualECGFormer, achieving an accuracy of 0.95, precision of 0.98, recall of 0.97, specificity of 0.82, and an *F*_1_-score of 0.97. DualECGFormer yielded slightly lower performance, with an accuracy of 0.92 and specificity of 0.63.

DualResNetECG demonstrated higher specificity and negative predictive value (NPV) than DualECGFormer for both PR interval and P-wave axis existence, reflecting improved identification of physiologically undefined parameters. Specificity increased from 0.60 to 0.81 for the PR interval and from 0.63 to 0.82 for the P-wave axis, while NPV improved from 0.69 to 0.79 and 0.68 to 0.79, respectively.

**Figure 7.**
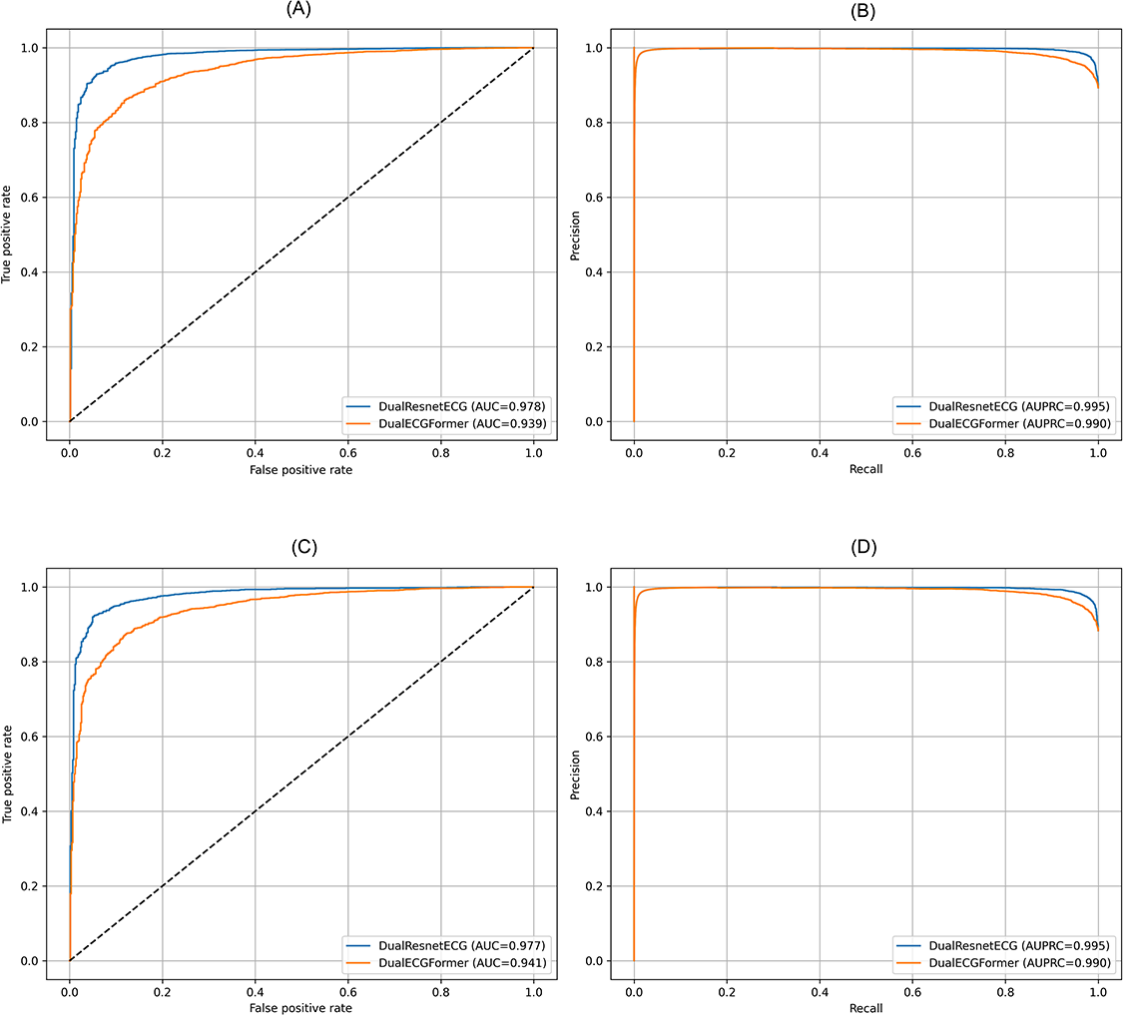
Receiver operating characteristic (ROC) and precision-recall (PR) curves comparing DualECGFormer and DualResNetECG for detecting the existence of the PR interval and P-wave axis. (A) ROC curve comparison for the PR interval. (B) PR curve comparison for the PR interval. (C) ROC curve comparison for the P-wave axis. (D) PR curve comparison for the P-wave axis. DualResNetECG achieved higher area under the receiver operating characteristic curve (AUROC) and area under the precision-recall curve (AUPRC) values for both parameters. AUC: area under the curve.

Consistent patterns were observed in receiver operating characteristic and precision-recall analyses (Figure 7). For PR interval existence, DualResNetECG achieved an area under the receiver operating characteristic curve (AUROC) of 0.978 and an area under the precision-recall curve (AUPRC) of 0.995, compared with an AUROC of 0.939 and an AUPRC of 0.990 for DualECGFormer. For P-wave axis existence, DualResNetECG achieved an AUROC of 0.977 and an AUPRC of 0.995, whereas DualECGFormer achieved an AUROC of 0.941 and an AUPRC of 0.990.

## Discussion

### Principal Findings

Vector-based ECG PDFs inherently preserve the geometric information of the original waveforms through embedded graphical path objects. By leveraging the Cartesian coordinates and drawing commands associated with these vector elements, it is possible to accurately reconstruct ECG time-series signals without reliance on raster image processing. In the present study, we demonstrated that the ECG waveforms encoded within vector-based PDFs can be restored to their original temporal and amplitude representations, confirming the feasibility of this approach for high-fidelity signal reconstruction. Our findings are consistent with previously published methods that used an intermediate PDF-to-SVG conversion step, wherein vectorized graphical elements within the SVG format are subsequently decoded to recover ECG signals [8]. The concordance between our results and those obtained using SVG-based approaches supports the validity of using vector graphics as a reliable source for ECG signal extraction. To the best of our knowledge, direct parsing of vector-based ECG PDFs for signal reconstruction—without the need for intermediate format conversion—has not been systematically described in the literature. The removal of intermediate processing steps contributes to a lighter and more scalable pipeline with computational efficiency, which is particularly advantageous for retrospective analyses of extensive clinical ECG repositories. Nevertheless, the proposed parsing framework was developed and validated exclusively using vector-based ECG PDFs generated by the MUSE system within a single health care institution. Consequently, the generalizability of the pipeline across ECG PDFs produced by other vendors remains to be further investigated.

Accurate estimation of ECG parameters—including ventricular rate, PR interval, QRS duration, QT and QTc intervals, and electrical axes—is indispensable for precise clinical diagnosis [21]. Our results demonstrated that both deep learning architectures, DualResNetECG and DualECGFormer, consistently outperformed the rule-based NeuroKit2 framework for most of the investigated parameters. While traditional rule-based algorithms offer high interpretability, they rely on deterministic signal processing chains characterized by derivative-based thresholding for peak detection and wavelet-based delineation for waveform boundaries [9,16,17]. As such, their performance is inherently dependent on predefined heuristics and signal quality assumptions, which may limit generalizability across heterogeneous clinical recordings [22].

In contrast, the superior performance of our deep learning models is rooted in their capacity for high-dimensional, nonlinear feature extraction, providing enhanced resilience against the intrinsic variability of clinical ECG recordings [23]. Specifically, the hierarchical representations in residual networks and the global contextual modeling of Transformer architectures effectively capture both localized waveform morphologies and complex interlead dependencies [18,19]. The modest performance lag of DualECGFormer relative to DualResNetECG may be attributed to the relatively short input duration (2.5 s per segment) in the 12-lead branch. This temporal constraint likely limits the efficacy of the Transformer’s self-attention mechanism, which typically thrives on more extended sequences. Conversely, the SE blocks in DualResNetECG facilitate explicit channel-wise feature recalibration, enhancing the model’s ability to selectively weight salient features across the dual-input fusion architecture. This adaptive recalibration proves particularly robust for filtering noise and accommodating morphological variations, outperforming rigid, threshold-based heuristics. Our findings align with the ongoing paradigm shift toward data-driven ECG interpretation, reaffirming that end-to-end deep learning frameworks can effectively circumvent the constraints of manual feature engineering [10,24].

A key methodological aspect of this study is the development of a multitask, multihead deep learning framework capable of simultaneously estimating multiple clinically relevant ECG parameters within a unified architecture [25]. Previous studies have largely focused on isolated prediction tasks, such as QT or QTc interval estimation, whereas the proposed DualResNetECG model enables concurrent prediction of ventricular rate, temporal intervals, and electrical axes [12-14,26-28]. The results indicate that this integrative design does not compromise predictive performance. DualResNetECG achieved low estimation errors for core temporal intervals, with MAEs of 7.27 milliseconds for the PR interval, 9.89 milliseconds for QRS duration, 11.87 milliseconds for the QT interval, and 13.71 milliseconds for QTc, alongside coefficients of determination ranging from 0.65 to 0.80. These findings suggest that shared representation learning within a unified framework can effectively capture the physiological relationships among diverse ECG parameters while maintaining reliable predictive accuracy.

Another important feature of this study is the explicit incorporation of “parameter existence” modeling alongside numerical estimation. This design enables the model to identify physiologically undefined parameters, which are commonly encountered in clinical practice. For the detection of PR interval and P-wave axis existence, DualResNetECG demonstrated high discriminative performance, achieving specificities of 0.81 and 0.82, respectively, with corresponding NPVs of 0.79 for both parameters. In addition, the model achieved AUROC values of 0.978 and 0.977 for PR interval and P-wave axis existence, respectively. These results indicate that the parallel classification head provides a reliable mechanism for distinguishing cases in which parameter estimation is not physiologically meaningful, such as in atrial fibrillation or junctional rhythms. By integrating physiological definability into the prediction framework, this approach enhances the clinical interpretability and reliability of automated ECG analysis without relying on post hoc rule-based corrections.

### Limitations and Future Directions

This study has several limitations that should be acknowledged. First, all ECG PDF files analyzed in this work were generated by the MUSE ECG management system. Consequently, the proposed PDF parsing and signal reconstruction approach was specifically tailored to the structural characteristics of MUSE-formatted documents. Although the methodology leverages the general principle that ECG waveforms are encoded as vector-based graphical path objects, its direct applicability to PDF files produced by other vendors has not been formally evaluated. Nevertheless, given that many ECG reporting systems use vector-based representations, the proposed framework is theoretically adaptable to other formats with appropriate modifications to accommodate differences in document layout, such as variations in lead arrangement and the positioning of individual leads and calibration signals. Future work should evaluate generalizability on PDFs generated by other vendors (eg, Philips and Mortara) using similar vector-path parsing principles.

Second, the ground truth labels used for training and evaluating the ECG parameter estimation models were derived from machine-generated measurements embedded within the ECG reports. Although these automated measurements are routinely generated and used within contemporary ECG management systems, they may contain inherent inaccuracies or systematic biases. Therefore, the proposed models should primarily be interpreted as systems designed to reproduce the automated outputs of the originating ECG management system rather than as direct substitutes for expert cardiologist adjudication or interpretation. Future studies could enhance label reliability by incorporating manual annotations or expert cardiologist adjudication. Additionally, expanding the size and diversity of the training dataset may further enhance the robustness and generalizability of the proposed models.

### Conclusions

We developed an integrated pipeline for reconstructing ECG time-series signals and estimating machine-generated ECG parameters directly from MUSE-generated vector-based ECG PDF reports. The method enables scalable digitization of archived ECG PDF records and supports simultaneous deep learning–based multitask prediction of 8 ECG parameters together with their corresponding physiological existence. The proposed approach achieved high reconstruction fidelity and predictive performance relative to the original MUSE-derived parameter outputs.

## Supplementary material

10.2196/80597 Multimedia Appendix 1 Representative example of the path object for lead I, consisting of 1238 coordinate points that define the discrete waveform extracted from the electrocardiogram (ECG) PDF. The x- and y-coordinate values are sample specific and may vary across different ECG records.

## Abbreviations

AUPRC: area under the precision-recall curve
AUROC: area under the receiver operating characteristic curve
CNN: convolutional neural network
CUDA: Compute Unified Device Architecture
ECG: electrocardiogram
GPU: graphics processing unit
IRB: institutional review board
MAE: mean absolute error
NPV: negative predictive value
QTc: corrected QT interval
SE: Squeeze-and-Excitation
SVG: Scalable Vector Graphics
