# Supplementary material for: Direct Reconstruction of High-Fidelity Electrocardiogram Signals From Vector-Based PDF Files With Integrated Deep Learning for Multiparameter Estimation: Retrospective Methodological Study
Source: JMIR Form Res. 2026 Jul 24;10:e80597. doi: 10.2196/80597 (PMC13399408; doi:10.2196/80597)
Supplement: Multimedia Appendix 1 [file formative-v10-e80597-s001.docx]

| **x** | **y** | **command** |  |  |
| --- | --- | --- | --- | --- |
| 1080 | 14,174 | M | ⇨Move to: | Start a path at the coordinates given |
| 1085 | 14,174 | L | ⇨Line to: | Draw a line segment from the previous point in the trail to the coordinates (1085, 14,174) |
| 1090 | 14,183 | L | ⇨Line to: | (1090, 14,183) |
|  | . |  |  |  |
|  | . |  |  |  |
|  | . |  |  |  |
| 7260 | 14,266 | L | ⇨Line to: | (7260, 14,266) |
| 7265 | 14,262 | L | ⇨Line to: | (7265, 14,262) |
|  |  | S | ⇨Stroke: | End of a path |
